# Derivatives of Esculentin-1 Peptides as Promising Candidates for Fighting Infections from *Escherichia coli* O157:H7

**DOI:** 10.3390/antibiotics11050656

**Published:** 2022-05-13

**Authors:** Raffaella Scotti, Bruno Casciaro, Annarita Stringaro, Fabrizio Morgia, Maria Luisa Mangoni, Roberta Gabbianelli

**Affiliations:** 1 Biological Service, Italian National Institute of Health, 00161 Rome, Italy; raffaella.scotti@iss.it (R.S.); fabrizio.morgia@iss.it (F.M.); 2 Laboratory Affiliated to Istituto Pasteur Italia-Fondazione Cenci Bolognetti, Department of Biochemical Sciences, Sapienza University of Rome, 00185 Rome, Italy; bruno.casciaro@uniroma1.it; 3 National Center for Drug Research and Evaluation, Italian National Institute of Health, 00161 Rome, Italy; annarita.stringaro@iss.it

**Keywords:** antimicrobial peptide, esculentin, biofilm, *Escherichia coli* O157:H7, antibacterial activity, antibiofilm

## Abstract

New strategies are needed to fight the emergence of multidrug-resistant bacteria caused by an overuse of antibiotics in medical and veterinary fields. Due to the importance of biofilms in clinical infections, antibiofilm peptides have a great potential to treat infections. In recent years, an increased interest has emerged in antimicrobial peptides (AMPs). One of the richest sources of AMPs is represented by amphibian skin. In the present work, we investigated the effects of two peptides derived from the frog skin AMP esculentin-1, namely, Esc(1-21) and Esc(1-18), on the growth, biofilm formation, and gene expression of the non-pathogenic *Escherichia coli* strain K12 and of enterohemorrhagic *E. coli* O157:H7. Both peptides showed minimal bactericidal concentrations ranging from 4 to 8 µM for Esc(1-21) and from 32 to 64 µM for Esc(1-18). They also, at sub-MIC doses, reduced the formation of biofilm, as supported by both microbiological assays and scanning electron microscopy, while they displayed no marked activity against the planktonic form of the bacteria. Transcriptional analysis in *E. coli* O157:H7 showed that both AMPs induced the expression of several genes involved in the regulation of formation and dispersal of biofilm, as well as in the stress response. In conclusion, we demonstrated that these AMPs affect *E. coli* O157:H7 growth and biofilm formation, thus suggesting a great potential to be developed as novel therapeutics against infections caused by bacterial biofilms.

## 1. Introduction

**Figure 1:**
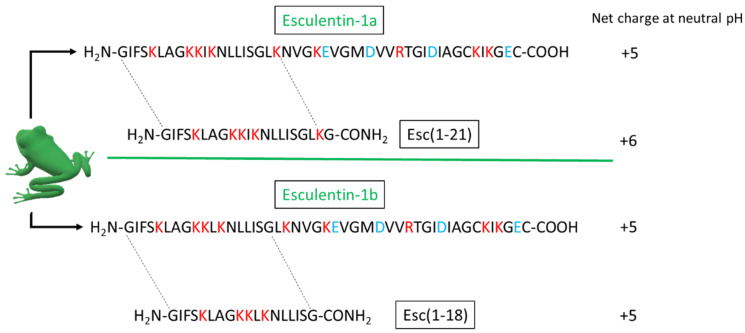
Primary structure and net charge of the two parent peptides from frog skin (esculentin-1a and esculentin-1b) and the two respective derivatives under investigation [i.e., Esc(1-21) and Esc(1-18)]. Basic and acidic residues are in red and light blue, respectively.

The emergence of drug-resistant bacteria and the difficulty in killing them are mainly associated with the aggregation of microorganisms within a self-produced matrix called a biofilm [1]. In their natural habitat, microorganisms exist prevalently in the form of biofilms, which are considered an adaptive response to environmental stress and confer bacteria with increased resistance compared to that of their planktonic counterparts. According to the National Institute of Health, it is estimated that 65–80% of all bacterial infections are due to biofilm-associated microorganisms [2]. The biofilm-related infections are highly resistant not only to common disinfectants, but also to conventional antibiotics that are generally used to kill planktonic bacteria, rendering biofilm infections extremely difficult to eradicate [3,4]. Enterohemorrhagic *Escherichia coli* O157:H7 (EHEC) is a human pathogen belonging to the attaching and effacing (A/E) *E. coli* group. It possesses virulence factors that are essential for adhesion to intestinal epithelial cells (attachment), and it is responsible for the destruction of the brush border of microvilli (effacement) [5]. The occurrence of these pathogens in the food chain is responsible for food-borne diseases. This pathogen can cause bloody diarrhea and hemorrhagic colitis, and approximately 4% of the cases develop into hemolytic uremic syndrome. The ability of *E. coli* O157:H7 to adhere to different biological surfaces to form sessile communities, the presence of persisting forms that cause severe food safety issues, and the absence of an effective therapy against EHEC infections have led to the need for new antimicrobial agents. Thus, the search for alternative strategies for treating and preventing bacterial infections is highly demanded. In recent years, antimicrobial peptides (AMPs) have attracted a growing interest as a therapeutic alternative to conventional anti-infective agents. Currently, there are more than 3100 experimentally reported AMPs [6]. AMPs are naturally occurring polypeptides containing cationic and hydrophobic amino acids (~12–50 residues) with direct antibacterial activity. AMPs are widely distributed in the animal and plant kingdoms, and they act to neutralize a broad range of microbes. Recently, many peptides have been identified with a prevalent biofilm inhibitory action [7,8,9]. More than a thousand AMPs arising from the skin secretions of amphibian species have been reported in the last few years. These peptides rapidly kill a broad range of microbes by perturbing their anionic plasma membrane and/or inactivating intracellular targets [10,11,12]. Esculentins-1 are a class of frog skin peptides with a highly conserved amino acid sequence consisting of 46 residues and characterized by a C-terminal loop [13,14]. Esculentin-1a and -1b differ by only one amino acid in position 11 of their sequence [15]. During the last few years, two peptides named Esc(1-21) and Esc(1-18), corresponding to the first 20 and 18 amino acids of the native esculentin-1a and -1b, respectively (Figure 1),were synthesized and found to display a large spectrum of bactericidal activity [14], encompassing the Gram-positive *Streptococcus agalactiae* and *Corynebacterium jeikeium* bacterial strains, the Gram-negative *E. coli* and *Pseudomonas aeruginosa* strains, and the yeast *Candida albicans* [16,17,18,19,20,21]. Considering the good activity of Esc(1-18) and Esc(1-21) on non-pathogenic reference *E. coli* strains [16,22], we assessed their antibacterial and antibiofilm activity against the bacterial pathogen *E.coli* O157:H7 EDL933 reference strain. We examined the effects of these two peptides on the expression of a set of genes implicated in the: (i) regulation of biofilm formation and dispersal; (ii) response to oxidative and osmotic stresses; (iii) stringent response, which is induced by nutritional stress conditions.

## 2. Results

### 2.1. Antibacterial Activity of the Peptides

The antimicrobial activity of the peptides was initially tested with a microdilution broth assay to determine the minimal inhibitory concentration (MIC). Esc(1-21) showed the strongest inhibitory effect on both the *E.coli* K12 and *E.coli* O157:H7 EDL933 strains with MICs of 2 and 4 µM compared to the higher values of 16 and 32 µM found for Esc(1-18), respectively (Table 1).

The antimicrobial activity of Esc(1-21) was also stronger than that of kanamycin, which was included as a reference antibiotic and showed a MIC of 16 µM against both strains [23]. We then evaluated the minimal bactericidal concentration (MBC) and found that it was equivalent to 2 × Mic for either the peptides or kanamycin.

**Figure 2:**
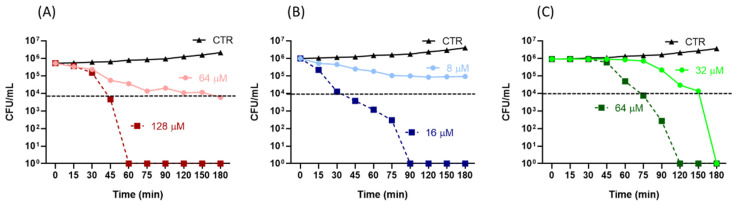
Time-kill assay of the *E. coli* O157:H7 EDL933 strain in the presence of Esc(1-18) (**A**), Esc(1-21) (**B**), and kanamycin (**C**). Concentrations of antimicrobial compounds were 2 × and 4 × Mic: 64 and 128 μM for Esc(1-18), 8 and 16 μM for Esc(1-21), and 32 and 64 μM for kanamycin. CTR indicates the controls. The dotted line represents a 2-log reduction (99%) of the initial inoculum. The data are the means ± standard deviations (SDs) of at least three independent experiments (although they are not visible).

The rate of bactericidal activity of Esc(1-21) and Esc(1-18) on the EDL933 strain was then investigated with a time-kill assay (Figure 2).

A 99% killing rate (corresponding to a 2-log reduction of viable bacterial cells (dotted line in Figure 2) was obtained within the first 30 min of treatment with Esc(1-21) at 4 × Mic or within the first 45 min of incubation with 4 × Mic of Esc(1-18). In comparison, a longer time (75 min) was needed for kanamycin at 4 × Mic.

### 2.2. Inner Membrane Permeation

**Figure 3:**
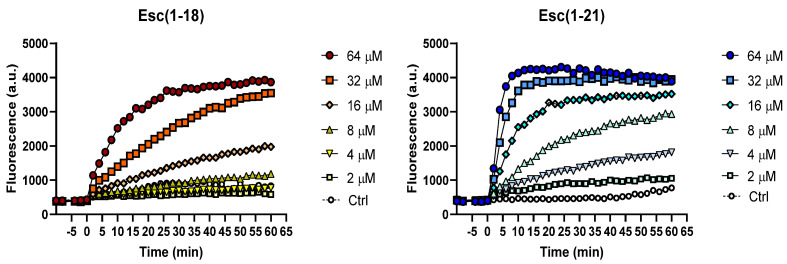
Membrane perturbation assay performed with Sytox Green dye in the presence of Esc(1-18) and Esc(1-21) on the EDL933 strain. Time 0 indicates the addition of the peptide. Data points are taken from a single experiment representative of three independent experiments. Controls (Ctrl) are cells not treated with peptides.

To evaluate the effects of the two peptides on the permeability of the inner membrane of the EDL933 strain, a Sytox Green assay was performed. Indeed, this allowed the assessment of the permeabilization of the bacterial cytoplasmic membrane by monitoring the increase in Sytox Green fluorescence upon its binding to intracellular nucleic acids. Sytox Green is a molecule that is impermeable to intact phospholipid bilayers, and the increase in fluorescence intensity is directly correlated to the membrane’s damage, which leads to the intracellular influx of the dye, followed by its binding to DNA. As reported in Figure 3, after peptide addition (time = 0), the increase in fluorescence intensity was dose dependent, with a more marked perturbation induced by Esc(1-21). In fact, low concentrations of Esc(1-18) (i.e., 4 and 8 μM) did not provoke any significant fluorescence change with respect to the control cells, while the greatest effect was recorded at the highest concentration of 64 μM within 60 min. This is in line with the membrane-perturbing activity that was previously observed for Esc(1-18) on the reference *E. coli* strain ATCC 25922 [17]. On the contrary, at the same low concentrations of 4 and 8 μM of Esc(1-21), a fluorescent signal was recorded within 5–10 min, while, at the highest concentration of 64 μM, the membrane-perturbing effect occurred within the first minutes after peptide addition.

### 2.3. Antibiofilm Activity

**Figure 4:**
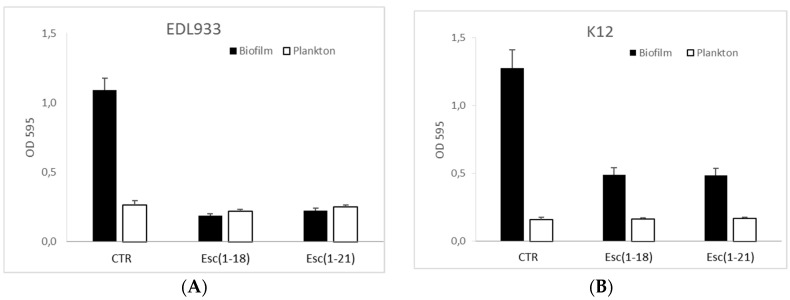
Antibacterial and antibiofilm activity of the peptides on *E. coli* strains. Planktonic growth (white bars) and biofilm formation (black bars) were quantified in modM9 (CTR) and in modM9 in the presence of 2 or 1 μM for Esc(1-21) and 16 or 8 μM for Esc(1-18) on the EDL933 (**A**) and K12 (**B**) strains, respectively. The bacterial culture was grown for 24 h in 96-well plates at 28 °C. The optical density (OD) of the planktonic cell suspension was measured at 595 nm to evaluate the bacterial turbidity before its removal from each well of the microtiter, while the OD of the biofilm was measured after CV staining of adherent cells at the same wavelength of 595 nm (see Section 4). Data are presented as mean ± SD.

To investigate the antibiofilm activity of the peptides, we initially analyzed their ability to affect biofilm formation at different concentrations. Figure 4 shows that both peptides at ½ MIC greatly inhibited the capacity of the EDL933 strain to form biofilm on polystyrene plates, while a weaker effect of the peptides was obtained towards the K12 strain.

Interestingly, both peptides turned out to be more potent in inhibiting biofilm formation rather than the growth of the planktonic form; in fact, the planktonic growth was not affected at the concentration used in the biofilm assay.

### 2.4. Effects of Peptides on Gene Expression

To understand the molecular mechanisms underlying the inhibition of biofilm formation in the EDL933 strain, we monitored the effects of the peptides (at ½ MIC) on the expression levels of some genes related to the formation and dispersion of biofilm, as well as to the stress response, through quantitative real-time PCR.

**Figure 5:**
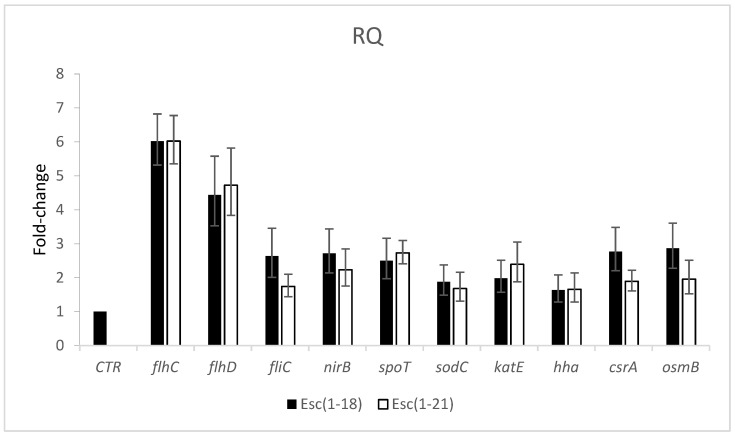
Expression levels of selected genes related to biofilm formation and stress conditions in the *E. coli* O157:H7 EDL933 strain treated with the peptides at sub-inhibitory concentrations of 2 and 16 μM for Esc(1-21) and Esc(1-18), respectively, compared to the untreated control samples (CTR). Transcriptional profiles were measured with qRT-PCR. *p* < 0.01 for all bars with respect to CTR.

The results shown in Figure 5 revealed a variable degree of upregulation of the microbial RNA of the *flh*C, *flh*D, *fli*C, *nir*B, *spo*T, *sod*C, *kat*E, *csr*A, and *osm*B genes in cells treated with Esc(1-18) and Esc(1-21) with respect to the untreated EDL933 strain.

The genes with the highest transcript abundance for both peptides were *flh*C and *flh*D; however, all genes that we examined showed a fold change greater than 1.5 fold.

Among the genes correlated with bacterial motility or its regulation, the *fli*C and *csr*A genes were upregulated by more than 2.6 and 1.7 fold in cells treated with Esc(1-21) and Esc(1-18), respectively, while the expression level of the *hha* gene increased by 1.6 fold for both peptides.

To study the potential effects of the peptides on the stringent response, we examined the expression levels of the *spo*T and *rel*A genes in the pathogen *E.coli* O157:H7 grown in a minimal medium. The results showed that both peptides induced an upregulation of *spo*T, but not of *rel*A, which was unchanged with respect to the control (data not shown). In addition, we observed an induction by more than two fold of the *nir*B gene, which encodes for the cytoplasmic nitrite reductase NirB, which is involved in the denitrification pathway of NO produced in the cytoplasm of bacteria. Furthermore, the genes involved in the response to stress conditions—oxidative or environmental stresses—were also upregulated. In fact, the *sod*C and *kat*E genes, which encode for the superoxide dismutase SodC and the catalase KatE, respectively, were over-expressed by more than 1.7 fold in the presence of the two peptides, indicating that they induced oxidative stress. Finally, the expression level of the *osm*B gene, which encodes for a lipoprotein involved in the osmotic balance’s maintenance, was two or three fold higher in the bacterial cells grown in the presence of Esc(1-21) or Esc(1-18) than in the cells grown in the absence of peptides.

### 2.5. Scanning Electron Microscopy (SEM)

**Figure 6:**
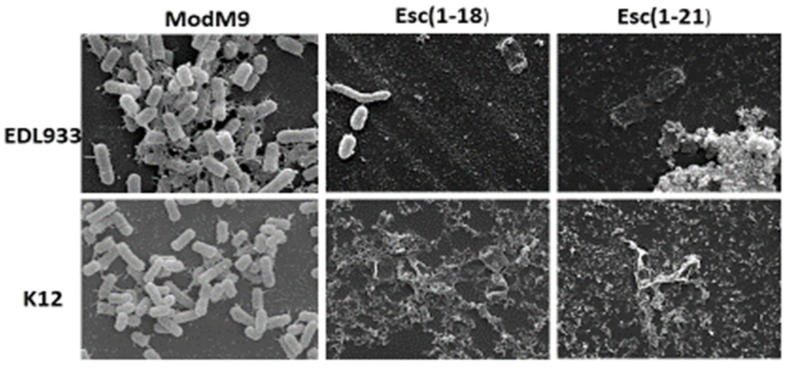
Effects of Esc(1-18) and Esc(1-21) on biofilm formation by *E. coli* strains. SEM was used to examine biofilm cells grown on glass coverslips in modM9 in the presence or absence of peptides at the concentration of ½ MIC for 24 h (magnification 30,000×).

We used SEM to observe the morphology of the biofilm of the EDL933 and K12 strains formed in the presence of each peptide at ½ MIC (Figure 6).

The results showed that the bacteria grown in the modM9 medium appeared numerous, had smooth surfaces, and were embedded in a dense matrix of polysaccharide material. In line with the results of the biofilm inhibition assay, in the presence of both peptides, only isolated cells were visible, and some showed a loss of turgidity, appearing flat, without connections, and lacking of a polymeric matrix. This could be ascribed to the impaired motility-to-adhesion switch required for biofilm formation. There were no substantial differences between the effects of the two peptides on both strains.

## 3. Discussion

In our study, we demonstrated the antibacterial activity of the Esc(1-18) and Esc(1-21) peptides against *E. coli* O157:H7, with Esc(1-21) appearing to be the most efficient one, as indicated by its lower MIC and MBC values. In addition, both peptides turned out to be stronger than kanamycin. This effect correlated with the killing kinetics, showing a significant reduction (99%) in viable bacterial cells within a short time (30 min) for Esc(1-21) or within 60 min for Esc(1-18) at 4 × Mic compared to 75 min for kanamycin. The results of the Sytox Green assay indicated the rapid perturbation of the cytoplasmic membrane by both peptides, with a more marked effect elicited by Esc(1-21), which was in agreement with the results of the time-kill curves (Figure 2), proving a stronger and faster bactericidal activity for Esc(1-21) with respect to Esc(1-18). This can be ascribed to the higher cationicity of Esc(1-21) compared to the isoform Esc(1-18) due to the additional basic residue at its C-terminal portion (Lys 20), which should increase the peptide’s interaction with the negatively charged phospholipids of the bacterial membrane. Previous circular dichroism experiments indicated an alpha-helical structure for both Esc(1-18) and Esc(1-21) in a membrane-mimicking environment [16,17]. The distribution of basic residues along the entire sequence of the peptides is expected to hamper their aggregation that is required for the formation of transmembrane pores, as described by the barrel–stave mechanism. Therefore, it is more likely that both Esc(1-18) and Esc(1-21) bind to the membrane surface in a carpet-like manner, inducing an unfavorable tension that would result in the formation of local membrane breakages leading to bacterial death, which is in line with previous results for Esc(1-18) on non-pathogenic *E.coli* [17]. In addition, we found that both Esc(1-21) and Esc(1-18) have the ability to prevent biofilm formation at a sub-inhibitory concentration (½ MIC), with Esc(1-21) being more potent than Esc(1-18). Moreover, both peptides turned out to be stronger inhibitors of biofilm formation than of planktonic growth; in fact, the planktonic growth of bacterial cells was not affected at the peptide concentration used in the antibiofilm assay.

To understand the role played by a series of genes related to biofilm formation/dispersal and to stress conditions, we examined their expression levels with real-time PCR and found an upregulation of all genes. In *E.coli*, motility is regulated by several global regulatory circuits that converge to modulate the overall expression of the master operon *flhDC* (class I flagellar operon) [24], which regulates the flagellar gene expression [25] and is controlled by a variety of physiological and environmental cues. This operon, in turn, regulates the class III flagellar gene (*fli*C), which encodes the major structural protein of the *E.coli* flagellum FliC. Flagella are known to have a crucial role in both early biofilm development and later biofilm dispersal. We demonstrated an increased expression of both the *flh*DC and *fli*C genes, indicating the genes that are involved in flagellar synthesis as targets of esculentins.

Two proteins associated with the *flh*DC operon, Hha and the global regulator CsrA, are involved in biofilm formation, and CsrA is also involved in biofilm dispersal. In *E.coli*, CsrA increases the expression of *flh*DC by directly binding to the mRNA to stabilize the transcript [24,26]. Sharma and colleagues [27] reported the positive interaction of Hha with the promoter of *flh*DC, demonstrating an Hha control of biofilm formation in *E.coli* O157:H7. We obtained a significant induction of *csr*A and *hha* in cells treated with Esc(1-21) and Esc(1-18), suggesting that, in the *E.coli* O157:H7 strain, esculentin-1 derivatives influence the *flh*DC operon through the induction of the *csr*A and *hha* genes. This suggests the plausible involvement of these genes in the peptide-mediated regulation of biofilm formation and dispersal.

It is known that a downregulation of the *flh*DC operon and *hha* gene is induced by the stringent response, an ubiquitous stress response found in microorganisms [28]. It is a response to nutritional stress, such as growth in a minimal medium, stimulating and inhibiting several biosynthetic pathways. It is characterized by the production of the signal molecules ppGpp and pppGpp—together referred to as (p)ppGpp—which regulate the expression of a plethora of genes promoting biofilm formation [29]. In bacteria, including *E.coli*, (p)ppGpp metabolism is driven by two genes: *rel*A and *spo*T. RelA produces the (p)ppGpp signaling molecules, as it possesses a synthetase functional domain, while SpoT regulates the degradation and production of (p)ppGpp, as it has both synthetase and hydrolase functional domains, with a strong hydrolase activity and a weak synthetase activity [29]. The (p)ppGpp molecule binds to the RNA polymerase, decreases transcription, and causes a global rewiring of the gene expression profile, leading to slow growth for most cells [30]. Casciaro and colleagues demonstrated the ability of Esc(1-21) and its diastereomer Esc(1-21)-1c to interact with (p)ppGpp [19], while, in another work [31], a specific interaction of a cationic peptide with (p)ppGpp was demonstrated, leading to the degradation of the nucleotide and the reduction of the available pool. More precisely, de la Fuente and coworkers hypothesized that AMPs blocked the widespread stringent response, thus reducing the ability of *E. coli* cells to form biofilm. In our study, we obtained an induction of *spo*T, but not of *rel*A (unchanged with respect to the control), and due to the prevalent hydrolase activity of SpoT, we hypothesized that the peptides lowered the level of the (p)ppGpp pool through its hydrolysis with the consequent inhibition of the stringent response and upregulation of the *flh*DC operon. It is known that in *E.coli*, flagella are important for reaching a suitable surface for adhesion. Once the surface is reached, other superficial cellular structures are important for adhesion, such as type I pili and curli [32,33], and flagella appear to be required neither for initial adhesion nor for further biofilm development. Motility and adhesion are two different and mutually exclusive bacterial characteristics, and both are under the control of regulatory cascades, each with a master regulator at the top. Pesavento and coworkers [34] provided evidence that in *E.coli*, the motility-to-adhesion switch requires a precise downregulation of motility. They found that the continued expression of the flagellar master regulator FlhDC strongly inhibits curli expression. The induced overexpression of the flagellar system highlighted in our study could be responsible for the inhibition of the cascade control of the synthesis of curli and the consequent inhibition of the adhesion and development of biofilm.

This effect was also visible through electron microscopy. Indeed, as shown in Figure 6, the untreated bacterial cells showed the presence of different types of fimbriae connecting the cells in the biofilm structure. On the contrary, peptide-treated cells were not able to form such communities, as indicated by the limited number of cells on the glass microscope slide and by the absence of a polymeric extracellular matrix, which was likely due to the impaired bacterial ability to adhere to surfaces, in line with both crystal violet (CV) staining (see Section 4 for antibiofilm activity) and gene expression analysis. In addition, some residual cells showed an altered morphological aspect, which was presumably due to the activation of the multi-stress response within 24 h of peptide treatment.

It is known that bacteria produce the toxic gas nitric oxide (NO) via the denitrification pathway for detoxifying nitrite produced in the cytoplasm. The *nir*BDC operon, which is positively regulated by nitrite, encodes for the cytoplasmic nitrite reductase NirB, which can reduce nitrite to NO [35]. This gas is identified as a key mediator of biofilm dispersal, and it can regulate biofilm dynamics in a variety of bacteria [36,37]. Recently, Park and colleagues [38], according to Barraud [39,40], found that in *P. aeruginosa* and in *E. coli* strains, NO stimulates biofilm dispersal or inhibits biofilm formation by affecting the motility. In our study, we observed that the *nir*B gene expression was induced in the presence of Esc(1-18) and Esc(1-21), suggesting a possible increase in NO with the consequent inhibition/dispersal of biofilm.

During their growth, bacterial cells encounter not only nitrosative stress, but also oxidative and osmotic stresses. Bacteria are killed by a variety of lethal stressors, some of which promote a cascade of reactive oxygen species (ROS). It is known that in *E.coli*, the presence of ROS activates the antioxidant defense system, which is represented by enzymes such as the superoxide dismutase SodC and the catalase KatE [41]. In the last few years, different works have demonstrated that antimicrobial drugs and AMPs induce the formation of ROS [42,43,44,45]. Charoenwong and colleagues [46] demonstrated that strains with mutations in the *osm*B gene, which encodes for a lipoprotein located in the outer membrane, were significantly more pressure sensitive than the wild-type strain. We investigated whether the selected peptides induced harmful levels of ROS and altered the osmotic balance by examining the transcription levels of *sod*C, *kat*E, and *osm*B in the *E.coli* O157:H7 strain. The higher expression that we observed for these genes after peptide treatment suggests the activation of a multi-stress response by the bacterial cells. This could lead to an altered bacterial morphology (as observed in Figure 6), which is presumably due to the osmotic imbalance within 24 h.

## 4. Materials and Methods

### 4.1. Peptides

Esc(1-18) and Esc(1-21) were purchased from Biomatik (USA/Canada). They were assembled through stepwise solid-phase synthesis and purified via reverse-phase high-performance liquid chromatography to a purity of >95% using a gradient of acetonitrile in 0.1% aqueous trifluoroacetic acid (from 28% to 100% in 30 min) at a flow rate of 1.0 mL/min. The molecular masses of each peptide were verified through electron spray ionization mass spectrometry.

### 4.2. Bacterial Strains

In this study, we used the enteropathogen *E. coli* O157:H7 reference strain EDL933 and the reference *E. coli* K12 MG1655 as a non-pathogenic strain. The cultures were obtained by inoculating modified M9 minimal medium (modM9), as described in an our previous work [47], with bacteria from fresh culture plates, and they were incubated at 28 °C overnight.

### 4.3. MIC and MBC Assays

The minimum inhibitory concentrations (MICs) were determined with the broth micro-dilution method [48]. The strains were grown in the modM9 with increasing concentrations of peptides. An overnight inoculum was added to the modM9 at the final concentration of 1 × 10^6^ CFU/mL in a total volume of 200 μL and in the absence or presence of peptides. Samples were inoculated in triplicate in a 96-well polystyrene plate (Becton Dickinson, Franklin Lakes, NJ, USA) and incubated at 28 °C with constant agitation for 24 h.

The MIC was determined as the lowest concentration of each peptide at which no visible growth was observed. Minimum bactericidal concentrations (MBCs) were obtained by streaking 100 µL from the clear wells of the microplate onto agar LB plates, which were incubated at 37 °C for 24 h. The MBC was defined as the lowest concentration of peptides at which no live bacteria were detected. To determine both the MICs and MBCs, we used peptide concentrations ranging from 1 to 16 µM for Esc (1-21) and from 8 to 128 µM for Esc(1-18). Kanamycin was used as a reference control [23].

### 4.4. Biofilm Formation Assay

A static biofilm formation assay was performed in 96-well polystyrene plates, as previously reported [49,50,51]. Briefly, overnight cultures were inoculated in modM9 in the absence or presence of peptides at an initial concentration of 1 × 10^6^ CFU/mL in a total volume of 200 µL, which was transferred into wells of a 96-well microplate and incubated at 28 °C for 24 h. Different peptide concentrations, ranging from ½ to 2 × MIC, were added to the cultures.

To quantify the total biofilm formation, the bacterial cell suspension was removed from each well of 96-well microplate after measuring its absorbance at 595 nm to evaluate the turbidity of the planktonic cells. Afterwards, each well was washed three times with PBS to remove non-adherent cells and air dried for 1 h. The biofilm was stained with 0.1% CV for 20 min and rinsed three times with H_2_O, and then the dye was dissolved in DMSO. The absorbance was measured again at 595 nm. The results are the average of at least 9 replicates.

### 4.5. Time-Kill Assay

The time-kill kinetics were determined for the peptides at concentrations of 8 and 16 µM for Esc(1-21) and at concentrations of 32 and 64 µM for Esc(1-18) (2 × and 4 × MIC, respectively). From a logarithmic growth phase, bacteria were added to modM9 at the final concentration of 1 × 10^6^ CFU/mL in a total volume of 200 μL in the presence or absence of peptides. Samples were inoculated in triplicate in a 96-well polystyrene plate (Becton Dickinson) and incubated at 28 °C for 24 h. A 100 µL aliquot was removed from each well, serially diluted, and plated on LB plates at 0, 15, 30, 45, 60, 75, 90, 120, 150, and 180 min. All plates were incubated at 37 °C for 24 h before the enumeration of the colonies. An antimicrobial compound is considered bactericidal if it kills ≥99.9% (3-log reduction) of the initial inoculum, as described by the CLSI [52]. The time-kill curve for the reference control kanamycin was determined at 32 and 64 µM for 2 × and 4 × MIC, respectively.

### 4.6. Permeation of the Bacterial IM

To verify the ability of Esc(1-18) and Esc(1-21) to perturb the cytoplasmic membrane of *E. coli* EDL933, the fluorescent probe Sytox Green was used as previously reported [21]. Approximately 1 × 10^7^ CFU/mL were incubated with 1 μM Sytox Green in PBS for 5 min in the dark for signal stabilization. After peptide addition, the fluorescence intensity (λ exc = 485 nm, λ ems = 535 nm) was monitored for 60 min in the microplate reader (Infinite M200, Tecan, Salzburg, Austria), and changes caused by the binding of the dye to intracellular DNA were recorded and plotted. The peptide concentrations ranged from 2 to 64 μM, and the controls were cells that were not treated with the peptides.

### 4.7. RNA Isolation and Quantitative Real-Time RT-PCR

To isolate the RNA of the EDL933 strain used in the real-time RT-PCR experiments, bacteria were inoculated in 8 mL of modM9 at the initial concentration of 1 × 10^6^ CFU/mL in the presence or in the absence of peptides, which were used at sub-lethal concentrations of 2 and 16 µM for Esc(1-21) and Esc(1-18), respectively. Cultures were incubated at 28 °C for 24 h with 250 rpm agitation and stabilized with RNA Protect Bacteria Reagent (Qiagen). RNA extraction was performed using a Presto Mini RNA Bacteria kit (Geneaid, New Taipei City, Taiwan), DNA contamination was eliminated using DNase I (Epicentre, Singapore) for 20 min at 37 °C, and RNA was precipitated with 0.7% isopropanol and 0.3 M sodium acetate. The absence of residual DNA was verified through PCR using specific primers. The quantity and the integrity of RNA were determined using a UV-VIS one-drop micro-volume spectrophotometer (DeNovix-Resnova, Wilmington, DE, USA) at 260 nm. RT-PCR was used to investigate the transcription levels of genes involved in the regulation of biofilm formation and dispersion (*csr*A, *hha*, *nir*B), in flagellar synthesis (*flh*C, *flh*D, *fli*C), in the stringent response (*rel*A, *spo*T), and in oxidative (*sod*C, *kat*E) and osmotic stress responses (*osm*B). To perform RT-PCR, an SYBR Green kit (Luna Universal One-Step RT-qPCR, BioLabs, Cambridge, MA, USA) was used. The PCR cycling conditions were as follows: cDNA synthesis at 55 °C for 10 min, a denaturation program at 95 °C for 1 min, and amplification and quantification were repeated 40 times: 95 °C for 10 s and 60 °C for 30 s. The specificity of PCR was determined with melting curve analyses (55 °C to 95 °C with a heating rate of 0.3 °C/s). Forward and reverse primers for the analyzed genes were designed using the Pel Primer software (Table 2).

To determine the efficiency of each primer pair, a series of five ten-fold dilutions were performed, and standard curves were generated. R2 values or correlation coefficients >0.95 were considered optimal correlations between values. The *16s* rRNA, a housekeeping gene, was used to normalize the levels of target gene expression detected between treated and untreated strains by measuring the changes in fold expression using the 2-ΔΔCT method [53]. Bacteria without treatments were used as a calibrator sample (control sample). All RT-PCR experiments were conducted at least in triplicate.

### 4.8. Scanning Electron Microscopy (SEM)

SEM was used to assess the morphological effects of peptides on the biofilm formed by *E. coli* in modM9. We used peptides at concentrations of 2 and 1 µM for Esc(1-21) and concentrations of 16 and 8 µM for Esc(1-18) for the EDL933 and K12 strains. Both untreated and treated biofilms formed on glass coverslips were obtained as described in our previous work [49]. Briefly, biofilms formed on coverslips of 12 mm diameter were fixed with 2.5% glutaraldehyde in sodium cacodylate buffer for 30 min and postfixed with 1% osmium tetroxide. These samples were dehydrated using a graded alcohol series and were critical-point dried in CO_2_ (CPD 030 Blazers device, Bal-Tec, Los Angeles, CA, USA). After being coated with gold by sputtering (SCD 040 Blazers device, Bal-Tec), the same samples were observed with an FEI Quanta Inspect FEG scanning electron microscope (FEI, Hillsboro, OR, USA).

### 4.9. Statistical Analysis

Experiments were performed in triplicate and repeated at least three times (*n* = 9). The results were presented as the average ± the standard deviation, and statistically significant differences (*p* < 0.01) were determined with Student’s *t*-test using the Excel software (Microsoft Office Excel 2016).

## 5. Conclusions

Our results mainly demonstrated that both Esc(1-18) and Esc(1-21) inhibit the biofilm formation of *E.coli* O157:H7. Based on the observation that the genes controlling the flagellar system of this bacterium are upregulated upon peptide treatment, we speculate that the inhibition of biofilm formation is likely due to the increased expression of these genes. In turn, this upregulation would be responsible for the inhibition of the cascade control of curli production with consequent inhibition of bacterial adhesion to surfaces and development of biofilm. Furthermore, our data have indicated that these peptides are able to influence the bacterial stress response, possibly through the production of ROS and by provoking an osmotic imbalance. Further studies for better understanding the mechanism of action will concern the design of knockout mutants of the genes of interest and the evaluation of the resulting phenotype.

## Figures and Tables

**Table 1:** Minimum inhibitory and bactericidal concentrations of Esc(1-18), Esc(1-21), and kanamycin against *E. coli* strains. The results are expressed in μM concentration and are derived from three independent experiments.

Strain	Esc(1-18)		Esc(1-21)		Kanamycin	
**EDL933**	MIC	32	MIC	4	MIC	16
	MBC	64	MBC	8	MBC	32
**K12**	MIC	16	MIC	2	MIC	16
	MBC	32	MBC	4	MBC	32

MIC = Minimal inhibitory concentration, MBC = Minimal bactericidal concentration.

**Table 2:** Primers used in this study for reverse transcription–quantitative PCR.

Oligo Name	Sequence 5′-3′	
*16s*	For	Rev
	CATCCACAGAACTTTCCAGAG	CCAACATTTCACAACACGAG
*csr*A	For	Rev
	TTAGTAACTGGACTGCTGGG	GTTGGTGAGACCCTCATGAT
*flh*C	For	Rev
	CGACTGGTTTATGACTTGGG	CTGGTGAGCGTGGGTAATAA
*flh*D	For	Rev
	CCGAGTTGCTGAAACACATTTA	ATTTATGCCGAGACGAAACA
*fli*C	For	Rev
	TTGCCGACTATACAGTCTCTTAC	TTGGTAGTGGTGTTGTTCAG
*Hha*	For	Rev
	AGGAAGGGATCTTGTCGTACAG	TCGTTGCCAGACAATTGACAC
*kat*E	For	Rev
	GTATTCATACCTTCCGCCTG	GTGCTTCATCCCAAACGAG
*nir*B	For	Rev
	TTACCTCGACGAAAGCAAAG	GCAGTTTATCAACACCGATAGA
*osm*B	For	Rev
	GTTCTAACTGGTCTAAACGGG	CCTAATGTACCCAACGTACTG
*relA*	For	Rev
	GATTACTGCTTCCGTTATCTCC	CGACCATACACTTCAGCTTTA
*sod*C	For	Rev
	GTCGAGATGAACCTCGTCAG	TCCAGACCTTTATCGGTTTCA
*spo*T	For	Rev
	ATGGCTGTGGAATGGGATAA	TCTTTCTCTTCCGTATTCAAACT

## Data Availability

Not applicable.
